# Supplementary material for: Genome-wide identification and expression analysis of the OSC gene family in Platycodon grandiflorus
Source: PeerJ. 2024 Oct 28;12:e18322. doi: 10.7717/peerj.18322 (PMC11639181; doi:10.7717/peerj.18322)
Supplement: Supplemental Information 5 [file peerj-12-18322-s005.docx]

**MIQE Checklist**

**1. Plant materials and treatment methods**

*Platycodon grandiflorus*, grown in pots and growing normally for one years, was used as the experimental material. The roots, stems, leaves, and root beard of *Platycodon grandiflorus plants* were separately collected to analyze the expression levels of the *PgOSC* genes in different tissues. To investigate the expression patterns of the *PgOSC* genes under various stress conditions, one-year-old *Platycodon grandiflorum* plants were subjected to salt stress (100 mM NaCl), heat stress (37°C for 2 hours), and drought stress (2 weeks). Subsequently, the expression level of the *PgOSC* genes were analyzed to assess its response to these stress environments. The sample was quickly frozen in liquid nitrogen and stored at -80 ℃. Each sample consists of three distinct plant tissues, and three experiments were conducted on them.

**2.Nucleic acid extraction and Reverse transcription**

The collected plant tissue samples are ground into powder in liquid nitrogen.Total RNA of each sample was extracted by using Trizol reagent (Takara, Beijing, China), RNase is not allowed to exist in the operating environment, reagents, and consumables. The nucleic acid tester (D3024R, Eppendoef, Germany)were used to determine the purity and concentration of RNA. RNA integrity was verified by 1.2% agarose gel electrophoresis. The first strand of cDNA was obtained by the reverse transcribing of 2 µg of RNA according to the First-Strand cDNA Synthesis Kit (Vazyme, Nanjing, China).The reverse transcription product was diluted 20 times and used for expression level analysis.

**3.Reaction system and reaction conditions of qPCR**

The reaction consisted of 10 µL SYBR Green Master Mix (Vazyme, Nanjing, China), 0.1 µmol L^-1^ primers F and primers R(primers used for qRT-PCR are listed in Table 1), 5.0 µL cDNA, and up to 20 µL with ddH_2_O. Bio-Rad CFX96 was used for qPCR and reaction conditions were set as follow: 95 ℃ for 3 min, 40 cycles of 95 ℃ for 10 s, 60 ℃ for 30 s, and 60 ℃ for 30 s. The dissolution curve procedure was set as: starting temperature is 60℃; final temperature is 95℃; holding time is 00:01; temperature increment is 0.2℃.

**4.Data analysis**

The Cq value were determined using the default threshold method, and the Cq values for each sample are shown in Table 2. Calculate the relative expression level of genes using the 2^- ∆∆ CT^ method. Repeat the experiment three times for each sample.

**Table 1. The list of primers used for qRT-PCR.**

| **Gene name** | **Forward (5'-3')** | **Reverse (5'-3')** |
| --- | --- | --- |
| *PgOSC2* | CGTCAGAATTATTCGCAGAC | ATTCCCCAATAGCCGTACCA |
| *PgOSC3* | GCATGTTGAGGTTACTTCATGT | TCGTTGGGATAAATGTCTGCTT |
| *PgOSC5* | CTCTACTTCACCTCTCAAAACTAC | CTTCTGTAGCATTTAGAAGCTCG |
| *PgOSC7* | GTACACACTGCATGGGCCAT | CCTGCCATTGGTAGTCCTGTTG |
| *PgOSC8* | GACATCGTCATTGAGCATGAGT | GTCCTCGAGGTACCTAGCAGC |
| *PgOSC9* | GCTATATCCTGGGCATAGAGAG | CCCATCCACCATCTTCCCG |
| *PgOSC11* | CCTGTCCAAGTTGCGCGAG | CCATCCTCTGCAAGCCATAAG |
| *PgOSC13* | CGATGGATCATGGTACGGTTGTT | CAGGTAGTTGCCTTGCTAGC |
| *PgOSC14* | CCGGTGGTTGGGGAGAAAGTT | GGTGCAATGGTGTCGGGTC |
| *PgOSC15* | CCACTGCATCGTGCAGC | GATTGAGGTACTCTCCGAG |
| *Pgactin* | CCGTGGAGCCAAGGGTTG | GGAGCACCCAAGCTTGCG |

**Table 2. The Cq values of qRT-PCR of *PgOSC* genes**

| Gene name | replication | Leaf | Steam | Root | Root beard |  | Control | Salt | Heat | Drought |
| --- | --- | --- | --- | --- | --- | --- | --- | --- | --- | --- |
| PgOSC2 | R1 | 20.81 | 21.26 | 17.68 | 18.80 |  | 22.56 | 22.29 | 22.41 | 22.35 |
|  | R2 | 21.11 | 21.63 | 17.60 | 20.63 |  | 22.86 | 22.40 | 21.43 | 21.72 |
|  | R3 | 20.71 | 20.72 | 18.14 | 20.33 |  | 22.70 | 22.24 | 21.65 | 22.03 |
| PgActin | R1 | 20.87 | 21.17 | 18.51 | 18.54 |  | 22.56 | 23.22 | 22.26 | 22.03 |
|  | R2 | 21.02 | 21.18 | 18.59 | 18.96 |  | 22.71 | 23.14 | 22.11 | 22.10 |
|  | R3 | 20.91 | 21.37 | 18.97 | 19.07 |  | 22.84 | 23.06 | 21.91 | 22.19 |
| PgOSC3 | R1 | 20.25 | 20.45 | 21.05 | 19.05 |  | 21.45 | 21.60 | 21.25 | 21.95 |
|  | R2 | 19.98 | 19.24 | 20.68 | 19.74 |  | 24.29 | 21.42 | 21.32 | 21.68 |
|  | R3 | 19.09 | 19.44 | 18.61 | 19.56 |  | 22.31 | 21.85 | 22.00 | 21.29 |
| PgActin | R1 | 20.09 | 19.62 | 21.37 | 19.01 |  | 21.45 | 21.91 | 21.68 | 21.91 |
|  | R2 | 20.01 | 19.21 | 21.33 | 19.15 |  | 21.12 | 21.83 | 21.96 | 21.84 |
|  | R3 | 19.37 | 19.26 | 19.18 | 18.99 |  | 22.31 | 21.85 | 21.93 | 21.93 |
| PgOSC5 | R1 | 19.10 | 18.96 | 18.90 | 19.28 |  | 20.37 | 19.65 | 21.65 | 21.38 |
|  | R2 | 18.92 | 19.57 | 18.87 | 18.67 |  | 20.83 | 19.46 | 21.08 | 21.61 |
|  | R3 | 18.83 | 18.36 | 18.72 | 19.55 |  | 20.10 | 19.26 | 22.39 | 21.54 |
| PgActin | R1 | 19.11 | 18.97 | 19.54 | 19.28 |  | 20.37 | 20.26 | 21.65 | 22.10 |
|  | R2 | 18.92 | 18.95 | 19.57 | 18.66 |  | 20.60 | 20.48 | 21.62 | 21.91 |
|  | R3 | 18.99 | 18.80 | 19.23 | 18.95 |  | 20.31 | 20.23 | 22.16 | 21.96 |
| PgOSC7 | R1 | 19.81 | 19.14 | 19.64 | 20.95 |  | 24.57 | 20.74 | 21.98 | 23.29 |
|  | R2 | 19.38 | 18.88 | 19.32 | 20.65 |  | 24.38 | 20.72 | 21.82 | 23.22 |
|  | R3 | 20.85 | 18.63 | 19.46 | 21.38 |  | 24.39 | 21.41 | 22.12 | 22.49 |
| PgActin | R1 | 19.30 | 18.99 | 19.56 | 20.22 |  | 24.37 | 21.71 | 21.74 | 23.29 |
|  | R2 | 19.39 | 18.73 | 19.71 | 20.14 |  | 24.38 | 21.79 | 22.16 | 23.21 |
|  | R3 | 19.12 | 19.01 | 19.84 | 20.06 |  | 24.57 | 22.17 | 22.27 | 22.57 |
| PgOSC8 | R1 | 19.25 | 19.22 | 18.17 | 18.74 |  | 22.82 | 24.56 | 22.23 | 21.98 |
|  | R2 | 19.11 | 19.71 | 17.82 | 18.52 |  | 22.49 | 24.23 | 23.03 | 22.12 |
|  | R3 | 18.76 | 19.71 | 18.78 | 19.04 |  | 22.39 | 22.09 | 23.12 | 22.76 |
| PgActin | R1 | 19.26 | 19.03 | 18.45 | 18.91 |  | 22.82 | 24.57 | 22.21 | 22.31 |
|  | R2 | 19.11 | 19.10 | 18.12 | 18.83 |  | 22.41 | 24.53 | 22.35 | 22.12 |
|  | R3 | 18.91 | 19.19 | 19.31 | 18.85 |  | 22.46 | 22.38 | 22.19 | 22.91 |
| PgOSC9 | R1 | 18.95 | 19.05 | 16.48 | 16.93 |  | 23.05 | 22.82 | 23.80 | 23.39 |
|  | R2 | 18.69 | 19.08 | 17.06 | 17.44 |  | 23.42 | 23.17 | 23.88 | 22.66 |
|  | R3 | 18.93 | 18.34 | 16.60 | 16.69 |  | 23.27 | 23.01 | 22.97 | 22.34 |
| PgActin | R1 | 18.68 | 18.91 | 17.37 | 17.26 |  | 23.04 | 23.26 | 23.11 | 23.39 |
|  | R2 | 18.96 | 18.84 | 17.60 | 17.48 |  | 23.42 | 23.46 | 23.23 | 22.65 |
|  | R3 | 18.93 | 18.93 | 17.31 | 17.23 |  | 23.34 | 23.31 | 23.30 | 22.32 |
| PgOSC11 | R1 | 18.64 | 18.65 | 19.96 | 22.01 |  | 23.51 | 21.83 | 22.82 | 23.13 |
|  | R2 | 18.77 | 18.19 | 20.37 | 21.21 |  | 23.26 | 21.89 | 22.44 | 21.88 |
|  | R3 | 19.02 | 18.96 | 20.27 | 21.78 |  | 22.89 | 22.24 | 22.40 | 21.99 |
| PgActin | R1 | 18.65 | 19.10 | 20.87 | 21.17 |  | 23.51 | 23.05 | 23.13 | 23.13 |
|  | R2 | 18.62 | 18.91 | 21.02 | 21.18 |  | 23.11 | 22.88 | 23.11 | 22.17 |
|  | R3 | 19.16 | 18.96 | 20.91 | 21.37 |  | 23.03 | 23.16 | 23.04 | 22.40 |
| PgOSC13 | R1 | 18.52 | 18.56 | 19.45 | 19.30 |  | 22.43 | 20.83 | 24.11 | 23.11 |
|  | R2 | 18.59 | 19.47 | 19.02 | 18.90 |  | 22.06 | 22.68 | 23.61 | 25.34 |
|  | R3 | 18.81 | 19.27 | 18.60 | 18.63 |  | 22.02 | 22.74 | 23.71 | 24.99 |
| PgActin | R1 | 18.51 | 18.54 | 20.09 | 19.62 |  | 22.11 | 22.03 | 23.96 | 23.76 |
|  | R2 | 18.59 | 18.96 | 20.01 | 19.21 |  | 22.06 | 23.45 | 23.90 | 25.67 |
|  | R3 | 18.97 | 19.07 | 19.37 | 19.26 |  | 22.28 | 23.42 | 23.71 | 25.82 |
| PgOSC14 | R1 | 21.36 | 19.16 | 18.96 | 19.66 |  | 25.71 | 25.28 | 23.13 | 22.15 |
|  | R2 | 21.52 | 19.33 | 18.65 | 20.58 |  | 26.05 | 22.48 | 22.36 | 23.45 |
|  | R3 | 18.92 | 19.66 | 18.98 | 19.04 |  | 25.91 | 22.40 | 22.44 | 23.05 |
| PgActin | R1 | 21.37 | 19.01 | 19.11 | 18.97 |  | 25.71 | 26.17 | 23.77 | 22.87 |
|  | R2 | 21.33 | 19.15 | 18.92 | 18.95 |  | 25.97 | 23.31 | 22.34 | 23.89 |
|  | R3 | 19.18 | 18.99 | 18.99 | 18.80 |  | 25.98 | 23.39 | 22.76 | 23.81 |
| PgOSC15 | R1 | 19.16 | 18.95 | 18.67 | 19.06 |  | 22.79 | 28.18 | 22.48 | 21.97 |
|  | R2 | 19.57 | 18.50 | 18.68 | 19.34 |  | 23.42 | 22.32 | 22.04 | 23.16 |
|  | R3 | 19.74 | 18.95 | 18.35 | 20.33 |  | 23.53 | 22.40 | 22.75 | 23.83 |
| PgActin | R1 | 19.54 | 19.28 | 19.30 | 18.99 |  | 23.17 | 25.17 | 22.77 | 22.87 |
|  | R2 | 19.57 | 18.66 | 19.39 | 18.73 |  | 23.42 | 22.31 | 22.34 | 23.89 |
|  | R3 | 19.23 | 18.95 | 19.12 | 19.01 |  | 23.01 | 22.39 | 22.76 | 24.82 |
